# The Dorsal Anterior Cingulate Cortex Modulates Dialectical Self-Thinking

**DOI:** 10.3389/fpsyg.2016.00152

**Published:** 2016-02-11

**Authors:** Fei Wang, Kaiping Peng, Yang Bai, Rui Li, Ying Zhu, Pei Sun, Hua Guo, Chun Yuan, Pia Rotshtein, Jie Sui

**Affiliations:** ^1^Department of Psychology, Tsinghua UniversityBeijing, China; ^2^Department of Biomedical Engineering, School of Medicine, Tsinghua UniversityBeijing, China; ^3^Department of Psychology, University of California, Berkeley, BerkeleyCA, USA; ^4^Center for Biomedical Imaging Research, Tsinghua UniversityBeijing, China; ^5^Department of Psychology, Peking UniversityBeijing, China; ^6^School of Psychology, University of BirminghamBirmingham, UK; ^7^Department of Experimental Psychology, Oxford UniversityOxford, UK

**Keywords:** naïve dialecticism, dialectical self-thinking, trait-judgment, functional magnetic resonance imaging, dorsal anterior cingulate cortex

## Abstract

Dialectical self-thinking involves holding the view that one can possess contradictory traits such as *extraverted* and *introverted*. Prior work has demonstrated that the dorsal part of anterior cingulate cortex (dACC) plays a crucial role in conflict monitoring as well as self-related processing. Here, we tested the function of dACC in dialectical self-thinking using a modified classical self-referential paradigm (self- vs. other-referential thinking), in which participants had to make a judgment whether a simultaneously presented pair of contradictory or non-contradictory traits properly described them while brain activity was recording using functional magnetic resonance imaging (fMRI). The data showed that activity in the dACC during the processing of self-relevant conflicting information was positively correlated with participants’ dispositional level of naïve dialecticism (measured with the Dialectical Self Scale). Psychophysiological interaction (PPI) analyses further revealed increased functional connectivity between the dACC and the caudate, middle temporal gyrus and hippocampus during the processing of self-relevant conflicting information for dialectical thinkers. These results support the hypothesis that the dACC has a key role in dialectical self-thinking.

## Introduction

Humans live in a world that can be full of contradiction. One strategy for dealing with contradictory information that has a long history in philosophy, but a relatively short history in psychology, is so-called ‘dialectical thinking.’ Piaget (1974) described dialectical thinking as the most sophisticated mental operation, extending far beyond formal operations in the development of thought processes. Dialectical thinking has been studied in a range of contexts, from assessments of personal beliefs about the world (ontologies) through to beliefs about the nature of knowledge (epistemologies; e.g., Basseches, 1979, 1984). Peng and Nisbett (1999) proposed three characteristics of naïve dialecticism: (i) *the principle of change*: everything is in constant dynamic flux; (ii) *the principle of contradiction*: contradiction exists everywhere, and contradictory aspects can coexist in the same thing; and (iii) *the principle of relationships or holism*: nothing is isolated, and all things are connected.

Previous studies in dialectical thinking have demonstrated the effect of naïve dialecticism in various cognitive domains, such as perception, attention, self-processing, emotion, and attribution judgments (Peng and Nisbett, 1999; Nisbett et al., 2001; Spencer-Rodgers et al., 2010). In these studies, East Asians have been more likely to manifest a dialectical thinking style compared with North Americans, for example, in making attributions (Morris and Peng, 1994; Choi and Nisbett, 1998), in reasoning about contradiction (Peng and Nisbett, 1999), and in predicting change (Ji et al., 2001). When reflecting personality traits in reference to the self, Chinese individuals are more likely to judge that pairs of contradictory traits (e.g., ‘*extraverted*’ and ‘*introverted*’) fit with themselves, while Americans are more likely to judge that only one of the pair can properly describe themselves (Spencer-Rodgers et al., 2004, 2009; Boucher et al., 2009). It should be noted that, although the concept of naïve dialecticism originated from cross-cultural research, it can also be measured as an individual difference variable within a single cultural group (English and Chen, 2007; Spencer-Rodgers et al., 2004, 2009), or be temporally manipulated using priming (Ma-Kellams et al., 2011).

Although the phenomenon of dialectical thinking has been well-established in social and cultural psychology and supported by a good deal of behavioral data, it remains unknown which brain region(s) govern this specific thinking style. In the current study, we focus on the manifestation of dialectical thinking in the domain of the self and attempt to identify the neural basis of this thought process. This investigation allowed us to assess how the brain responds to contradictory personality traits associated with ourselves compared with other people using a dialectical thinking style.

There are two lines of neuroscience literature that are relevant to the current investigation. Firstly, neuroimaging studies in the field of self-related processing have consistently shown that self-referential thinking, such as evaluating personality traits in relation to the self (e.g., deciding whether personality traits such as ‘*kind*’ properly describe oneself), is associated with increased activity in the cortical midline structures such as ventromedial prefrontal cortex (vmPFC) and anterior cingulate cortex (ACC) compared to when similar evaluations are made to others (Craik et al., 1999; Northoff and Bermpohl, 2004; Northoff et al., 2006; Zhu et al., 2007; Chiao et al., 2009, 2010; Denny et al., 2012; Ma et al., 2014). In addition to the cortical midline structures, a widely distributed set of regions has also been linked with various aspects of self-related processing. For example, activity in the caudate has been suggested to reflect the rewarding aspects of the self (Enzi et al., 2009); the ventrolateral prefrontal cortex (VLPFC) is involved in the retrieval of self-related semantic memory (Martinelli et al., 2013), while the hippocampus is implicated in self-related episodic memory (Svoboda et al., 2006; Addis et al., 2012). In addition, the anterior insula is associated with interoceptive self-awareness (Craig, 2009), and the fusiform gyrus with self-face recognition (Platek et al., 2008; Ma and Han, 2012). However, the neural basis of the dialectical aspect of the self has not been examined.

Secondly, prior work in the field of conflict processing has reported that the dorsal part of the ACC (dACC) plays a key role in monitoring and resolving conflict during information processing (Botvinick et al., 2004; Carter and van Veen, 2007). For example, the dACC was activated in conflict detection (Carter et al., 1999; Carter et al., 2000; MacDonald et al., 2000), in resolution of conflicting responses in Stroop-like tasks (Posner and DiGirolamo, 1998), and in focusing attention on task-relevant stimuli while ignoring task-irrelevant stimuli (Weissman et al., 2005). In recent years, the dACC has also been linked with dealing with conflict in higher-level processes such as resolving cognitive dissonance (van Veen et al., 2009; Izuma et al., 2010; Jarcho et al., 2011). In addition to the dACC, other attention-related regions have also been found to be involved in conflict-processing, such as the dorsolateral prefrontal cortex (DLPFC) and the parietal cortex (Luks et al., 2007; Stern et al., 2007; Roberts and Hall, 2008). Nevertheless, it is still unclear whether the dACC is critical to monitor and resolve conflicting information in self-referential thinking.

Dialectical self-thinking, by its definition, is a special form of self-referential thinking that deals with conflicting information in the domain of the self. Since both self-thinking and conflict-processing have been repeatedly linked to the ACC, in particular its dorsal part, we propose that the dACC is critical too in dialectical self-thinking. One key feature of naïve dialecticism is that individuals possessing a dialectical thinking style do not only passively accept the existence of contradiction and leave it as it is, but also try to compromise the contradictory aspects, reorganizing and integrating them into a cohesive whole (Peng and Nisbett, 1999; Spencer-Rodgers et al., 2004). For example, when facing social contradictions, Chinese individuals prefer dialectical resolutions (e.g., addressing the issues from both sides and reconciling the conflict by compromising) over non-dialectical resolutions (e.g., finding exclusive fault with one side; Peng and Nisbett, 1999, Study 2). Furthermore, Chinese participants who read about two contradictory studies, compared to those who read about only one of the studies, express more beliefs that are intermediate across both studies (Peng and Nisbett, 1999, Study 5). Lastly, in the domain of the self, compared to European Americans, Japanese participants have been shown to exhibit higher simultaneous accessibility to contradictory self-aspects while maintaining similar levels of processing speed, indicating that they were not less uncertain about self in general (Spencer-Rodgers et al., 2009, Study 2). Although previous research on the dACC mostly focused on the non-dialectical approach of conflict resolution (i.e., accepting one side of the contradiction and rejecting the other), this dialectical approach requires even more continuous exposure to and deeper processing of both sides of the contradiction and integrating them at a more holistic level. Thus, we predicted that chronic dialectical thinkers would be more likely to utilize the dACC, which is involved in conflict-processing as well as self-processing, to incorporate conflicting information about oneself into a cohesive self-concept.

To test this hypothesis, we employed a modified version of the well-established self-referential thinking paradigm (Zhu et al., 2007; Chiao et al., 2009, 2010). In our paradigm, participants were presented a pair of personality traits (contradictory vs. non-contradictory pairs) and had to judge whether the pair described themselves or a public figure (self vs. other-referential thinking). During the task, brain activity was recorded by functional magnetic resonance imaging (fMRI). Participants’ level of naïve dialecticism was measured as a dispositional construct with the Dialectical Self Scale (DSS; Spencer-Rodgers et al., 2004). We predicted that the dACC would be more active for chronic dialectical thinkers during the processing of self-relevant conflicting information. Furthermore, their dACC would play a more central role in their self-processing network and modulate activity in other nodes, resulting in increased functional coupling between the dACC and functionally linked regions during self-related conflict processing. This prediction was assessed using psychophysiological interactions (PPI) analysis (Friston et al., 1997) which examines the relative changes of functional connectivity between brain regions across the different experimental conditions.

## Materials and Methods

### Participants

Twenty-seven Chinese college students (16 females; 19–30 years old, mean ± SD = 22.30 ± 2.35) participated in the experiment. All participants were right-handed and had normal or corrected-to-normal vision. Informed consent was obtained from all participants prior to the experiment according to procedures approved by the ethics committee of Center for Biomedical Imaging Research, Tsinghua University.

### Stimuli

We created a list of 428 traits adjectives based on previously used personality traits pools (Spencer-Rodgers et al., 2004; Zhang et al., 2006; Zhu et al., 2007). Among them, 128 pairs of traits were chosen based on the level of contradiction in meaning and valences rated by six Chinese and six American participants. These traits were classified into 16 groups, and two versions of 4-pair subsets were created for each group. In the *contradictory version*, the original trait pairs that were contradictory in meaning as well as in valence were retained (e.g., *intelligent-stupid*).^1^ In *non-contradictory* versions, traits were re-paired with another trait in the same group such that the new pairs were non-contradictory in meaning and had similar valence (e.g., *stupid – untrustworthy*). Sixteen contradictory subsets were used for the contradictory condition and 16 non-contradictory pairs for the non-contradictory condition, and were counter-balanced across conditions and participants.

### Procedure

Participants performed trait-judgment tasks in relation to the self or to a well-known public figure (i.e., Hu Jintao, who was the Chairman of the People’s Republic of China at the time). The stimuli were presented through an LCD projector onto a rear projection screen at the head end of the bore of the scanner. Participants viewed the screen through an angled mirror positioned attached above the head-coil. During each scan, participants had to make judgments on whether pairs of traits correctly described either him/her (the *self* condition) or the public (the *other* condition). Thus, there were four types of experimental blocks: *contradictory self*, *non-contradictory self*, *contradictory other*, and *non-contradictory other*. At the beginning of each block, a 22.6° × 2.7° cue sentence describing the current judgment task (self vs. other; e.g., ‘Please judge whether the words presented can describe yourself’) was presented for 2 s indicating the judgment target, followed by four experimental trials. On each trial, a pair of traits of 5.6° × 1.6° was simultaneously presented bilaterally for 2 s, followed by two consecutive 2 s response windows during which time participants had to respond to each of the traits, respectively. In each response display, a cue sentence describing the current response task (e.g., ‘Please judge whether the left word can describe yourself’) of 22.6° × 2.7° was presented in the center of the screen and the participant pressed the one of two keys to make a yes/no judgment. Each experimental condition was repeated four times in a scan and the order of conditions was counter-balanced using a Latin Square design. Five 26 s fixation periods were inserted after every four blocks, as well as at the beginning and end of a scan. Two scans of 537.5 s were obtained from each participant.

After the scan session, participants completed DSS (Spencer-Rodgers et al., 2004) which is a widely used self-report measure of dispositional naïve dialecticism with adequate psychometric properties. DSS uses a 7-point Likert-like scale with 1 = *Strongly Disagree* and 7 = *Strongly Agree* for 32 items (e.g., ‘*My world is full of contradictions that cannot be resolved*.’). For the current study, DSS’s Cronbach’s α coefficient was 0.73, indicating adequate reliability.

### Image Acquisition

Imaging data were acquired at the Center of Bio-Medical Imaging Research (CBIR), Tsinghua University. A Philips Achieva 3.0T TX system with a standard 8-channel head coil was used to acquire T2-wighted echo-planar images (EPI) blood oxygenated level dependent (BOLD) contrast. Forty transverse slices were acquired with 3 mm thickness with a plane resolution of 2.5 mm × 2.5 mm. We used a 2500 ms slice repetition time, 90° flip angle, and 35 ms echo time. The slices covered the most of the brain excepted for the inferior parts of the cerebellum. A High-resolution T1-weighted image was also acquired for each participant with 160 contiguous sagittal slices of 1 mm thickness and 8° flip angle. Time of repetition was 8.2 s and time of echo was 3.8 ms. The acquisition matrix was 256 mm × 256 mm with voxel size of 1 mm × 1 mm × 1 mm.

### Data Analysis

SPM8 (Wellcome Department of Cognitive Neurology, London, UK; www.fil.ion.ucl.ac.uk/spm) was used for data analysis.

#### Preprocessing

The functional images were realigned to the first scan to correct for head movement, spatially realigned and unwrapped to correct for interactions between movement artifacts and field inhomogeneities, and slice-timed. The six estimated movement parameters were used as covariates in the subsequent individual-level statistical analysis. Adjusted functional images were co-registered with structural images, and structural images were segmented into different tissue types, the outputs of which were normalized to MNI space using the DARTEL algorithm implemented in SPM8 to increase the accuracy of inter-subject alignment (voxel-size was transformed to 1.5 mm × 1.5 mm × 1.5 mm in this step). Finally, the normalized images were smoothed with a Gaussian kernel of 8 mm.

#### Definition of dACC Region of Interest (ROI)

We defined an anatomical ROI of the bilateral dACC with WFU Pickatlas toolbox (Maldjian et al., 2003) using the procedure described recently (Cascio et al., 2015), by firstly creating the union of Brodmann areas 24 and 32, as well as AAL masks of anterior, middle, and posterior cingulate (all dilated to 2 mm), then subtracting Brodmann areas 8 and 9 from this union, and finally restricting this ROI to a bounding box of [*x* = –16 to 16, *y* = 0 to 33, and *z* = 6 to 52].

We created contrasts for each of the four experimental conditions (with two variables: judgment type – self vs. other; trait type – contradictory vs. non-contradictory) at the individual level. These contrast images were then submitted to a group-level voxel-based 2 × 2 repeated-measure ANOVA within the dACC ROI. A gray matter mask was created by binarizing SPM’s prior probability gray matter map at a threshold of 0.2, and this was applied in this and all of the following group-level analyses. Statistical maps were thresholded at *p*_uncorr_ < 0.005 and the issue of multiple comparisons was addressed with a small-volume corrected threshold of *p*_FWE_ < 0.05. Furthermore, clusters passing a more liberal cluster-level threshold of *p*_uncorr_ < 0.05 were reported as trending results.

#### Correlational Analysis

To test the hypothesis that dACC’s activity in the processing of self-related contradiction was modulated by the dispositional naive dialecticism, we conducted correlational analysis between the DSS score and brain activity. Contrasts reflecting the interaction between contradiction and the self [*contradictory (self – other) – non-contradictory (self – other)*] were created for each participant, and signal intensity in these images was submitted to a group-level regression model within the dACC ROI, with DSS score as a predictor and gender and age as covariates of no interest. Statistical maps were thresholded at *p*_uncorr_ < 0.005 and multiple comparisons were addressed with a small-volume corrected threshold of *p*_FWE_ < 0.05. Furthermore, clusters passing a more liberal cluster-level threshold of *p*_uncorr_ < 0.05 were reported as trends.

#### Exploratory Whole-Brain Analysis

To verify the results in the ROI analyses, univariate whole brain analyses were conducted. We performed the 2 × 2 repeated-measures ANOVA (judgment type – self vs. other; trait type – contradictory vs. non-contradictory) and correlational analysis within the whole-brain, using a voxel-wise threshold of *p*_uncorr_ < 0.005, combined with a cluster-level threshold of *p*_FWE_ < 0.05. Furthermore, clusters passing a more liberal cluster-level threshold of *p*_uncorr_ < 0.05 were reported as trends.

#### Psychophysiological Interactions (PPI) Analysis

To further explore the dACC’s changes in functional connectivity with other regions during the processing of self-related contradiction, a PPI analysis was conducted (Friston et al., 1997). The ROI was defined as a sphere with 3 mm radius centered at the peak coordinate [–12 31.5 27] in the dACC identified from the above analyses. Time series of the ROI were extracted, and the PPI regressor was calculated as the scalar product of the time-course of activity in the seed region and the task time-course of [*contradictory (self – other) – non-contradictory (self – other)*]. The individual-level contrast images were subjected to a group-level one-sample *t*-test, and the modulatory role of the DSS score was examined with a multiple regression model controlling for participants’ gender and age. Statistical maps were thresholded at *p*_uncorr_ < 0.005 and a cluster-level threshold was set at *p*_FWE_ < 0.05. Furthermore, clusters passing a more liberal cluster-level threshold of *p*_uncorr_ < 0.05 were reported as trends.

## Results

### Behavioral Results

Participants made valid responses (i.e., made a response within the time window) on 94.4% of the trials during the encoding phase. To assess the effect of dialectical self-referential thinking, the ratio of double-‘yes’ trial (i.e., ratio of participant made ‘yes’ responses to both of the traits) was subjected to a 2 (judgment: self vs. other) × 2 (trait type: contradictory vs. non-contradictory) repeated measures ANOVA (see **Table 1**). The analysis yielded a significant main effect of judgment target, *F*(1,26) = 8.06, *p* = 0.01; participants made more double-‘yes’ responses on self- relative to other-referential judgment trials. The main effect of trait type was also significant, *F*(1,26) = 111.75, *p* < 0.01; participants made more double-‘yes’ responses on non-contradictory relative to contradictory trials. The interaction between these two factors was marginally significant, *F*(1,26) = 3.26, *p* = 0.08; Simple main effect analysis showed that, while the percentage of double-‘yes’ responses decreased significantly from non-contradictory trials to contradictory trials for both judgment target (*p*s < 0.01), the reduction was greater for the self-judgment, indicating that participants were more reluctant to attribute conflicting adjectives to self than other target. Correlational analyzes showed that DSS score was not significantly correlated with double-‘yes’ rate in any condition (|*r*| s < 0.15, *p*s > 0.44), nor with the interaction effect (*r* = –0.05, *p* = 0.81).

**Table 1:** Percentage of double-‘yes’ response in the behavioral task.

	Self	Other
Contradictory	10.45%	6.19%
	±15.13%	±5.89%
Non-contradictory	35.88%	27.80%
	±14.64%	±11.93%


### fMRI Results

#### ROI Analyses

A cluster covering the bilateral dACC first was identified. The ANOVAs with judgment (self vs. other) and type of trait (contradictory vs. non-contradictory) demonstrated a significant main effect of *self* > *other*, *k* = 2587, *p*_FWE_ < 0.01, peaking at [0 31.5 16.5], *Z* = 4.90. No suprathreshold cluster was found for the main effect of type of trait; there was no greater activity involved in contradictory relative to non-contradictory condition. We also did not observe a significant interaction between judgment and type of trait [*contradictory (self – other) > non-contradictory (self – other)*], indicating that the dACC was not especially engaged during the processing of self-related conflicting information in the whole sample.

**FIGURE 1:**
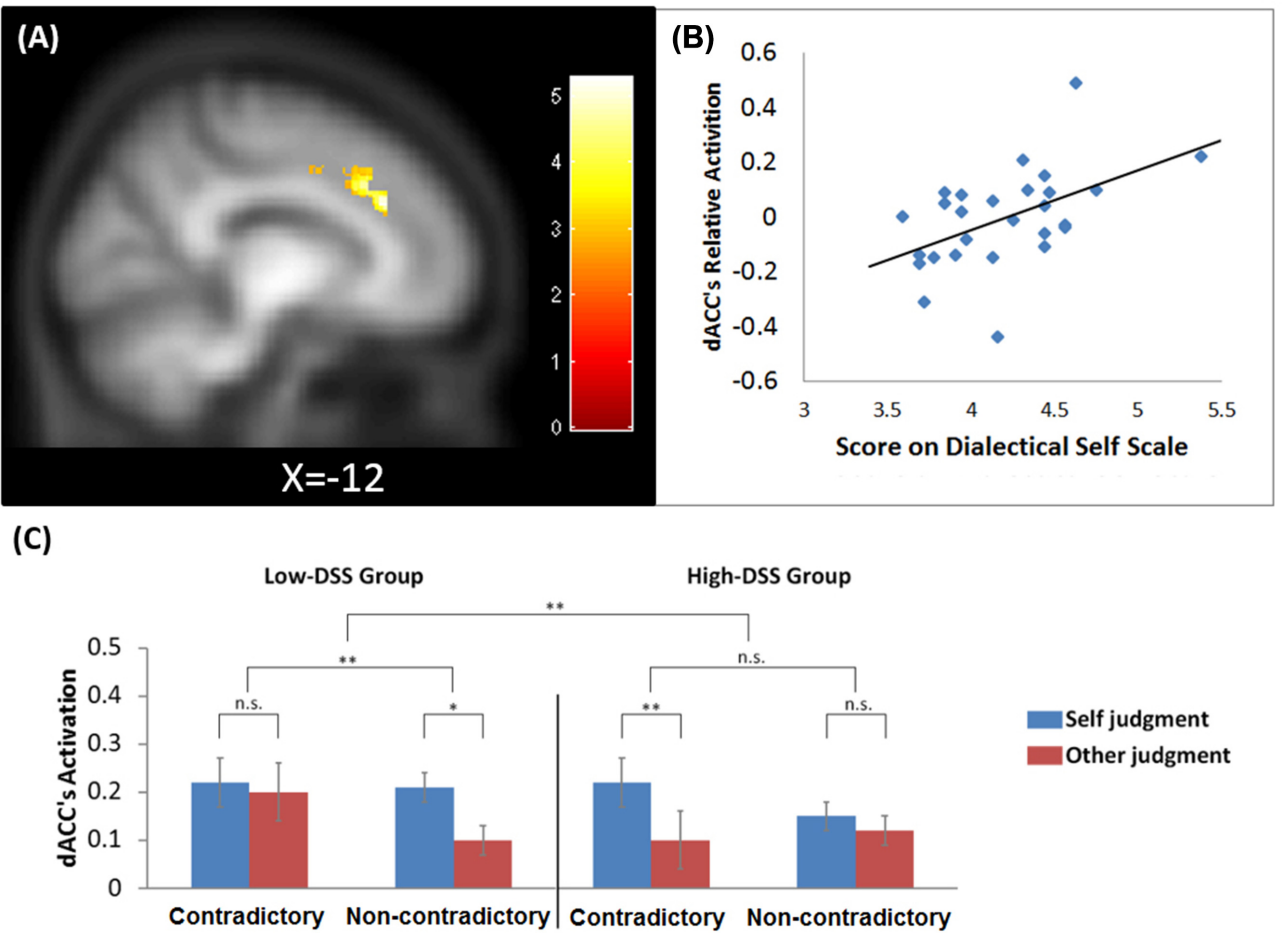
**(A)** A cluster in left dACC (peaking at [–12 31.5 27]) showed significant positive correlation between its relative activation during the processing of conflicting information relevant to self and score on Dialectical Self Scale (imposing on participants’ averaged smoothed anatomical images); **(B)** Scatter plot of the dACC’s relative activation and DSS score; **(C)** The dACC’s activation across conditions and DSS levels. ^∗^*p* < 0.05, ^∗∗^*p* < 0.01.

However, there was a significant positive correlation between the relative degree of activity of dACC during the processing of self-relevant information and the DSS score, *k* = 309, *p*_FWE_ < 0.01, peaking at [–12 31.5 27], *Z* = 4.22 (**Figure 1A**). The result indicated that the engagement of the dACC in the processing of self-related conflicting information was modulated by participants’ subjective dispositional level of dialectical thinking; the correlation coefficient between the cluster’s mean relative activation and the DSS score was *r* = 0.71, *p* < 0.01 (**Figure 1B**). Furthermore, to measure whether the strength of the DSS score modulated the strength of activity in dACC, participants were split into two groups using the mean DSS scores (the mean score for the high (*n* = 13) and low groups (*n* = 14) were 4.57 and 3.91, respectively). A mixed ANCOVA with three variables – trait type (contradictory vs. non-contradictory) × judgment (self vs. other) × DSS level (high vs. low), for gender and age, was conducted. We extracted the cluster’s mean activation in each condition using MarsBar 0.43 (Brett et al., 2002). The analyses showed a significant three-way interaction, *F*(1,23) = 18.42, *p* < 0.001. The follow-up analyses revealed that there was enhanced activity in the *non-contradictory* self-thinking condition for the low-DSS group; in contrast, there was increased activity in the *contradictory* self-thinking condition for the high-DSS group (**Figure 1C**).

#### Univariate Whole-Brain Analyses

As shown in **Table 2**, a voxel-based ANOVA performed on the whole brain verified the ROI results, consistent with prior work (Zhu et al., 2007), revealing a large cluster covering cortical midline structures such as the ACC and MPFC, generating a main effect of *self* > *other, k* = 11893, *p*_FWE_ < 0.01, peaking at [–3 57 –3], *Z* = 6.48. Two additional significant clusters were identified in left caudate and partial area, and trends for three additional clusters were identified in the right superior and middle frontal gyri, the bilateral SMA and SFG, and the right fusiform gyrus, lingual gyrus, and parahippocampus. The main effect of *contradictory* > *non-contradictory* identified a large cluster in the left parietal lobe, *k* = 6371, *p*_FWE_ = < 0.01, peaking at [–31.5 –48 66], *Z* = 4.64, as well as a trend for a cluster covering the left middle occipital gyrus. The contrast reflecting the interaction effect of *contradictory (self – other)* > *non-contradictory (self – other)* identified a cluster in the left hippocampus and parahippocampus, *k* = 1134, *p*_FWE_ = 0.01, peaking at [–24 –7.5 –24], *Z* = 4.81. Additionally, a cluster in the right superior and middle frontal gyrus was identified as trending region.

**Table 2:** Results of voxel-based ANOVA in exploratory whole-brain analysis.

Regions	BA	*k*	Peak			
			
			*x*	*y*	*z*	*Z*
*Self* > *Other*						
Bilateral ACC, MPFC, and MFG	9/10/24/32	11893^∗∗^	–3	57	–3	6.48
Bilateral caudate		1999^∗^	–18	22.5	9	4.43
Left calcarine, PCC, lingual, precuneus, parahippocampus, hippocampus	18/19/30/31	2190^∗∗^	–24	–61.5	18	4.40
Right SFG and MFG	10	817^†^	25.5	57	27	4.26
Bilateral SMA and SFG	6/8	1409^†^	10.5	18	61.5	3.95
Right fusiform, lingual, and parahippocampus	19	251^†^	30	–52.5	0	3.85
*Contradictory* > *Non-contradictory*						
Left IPL, SPL, MFG, SFG, postcentral, and precentral	3/5/6/7/9/40	6371^∗∗^	–31.5	–48	66	4.64
Left MOG		628^†^	–28.5	–76.5	19.5	3.71
*Contradictory (Self – Other) > Non-contradictory (Self – Other)*						
Left hippocampus and parahippocampus	28	1134^∗^	–24	–7.5	–24	4.81
Right MPFC, SFG, and MFG	10	684^†^	12	55.5	19.5	4.74
Left cerebellum and bilateral brain stem		771^†^	–4.5	–46.5	–15	3.63


*ACC, anterior cingulate cortex; MPFC, medial-prefrontal cortex; MFG, middle frontal gyrus; SFG, superior frontal gyrus; PCC, posterior cingulate cortex; SOG, superior occipital gyrus; MOG, middle occipital gyrus; SMA, supplementary motor area; IPL, inferior parietal lobule; SPL, superior parietal lobule; ^∗∗^*p*_*FWE*_ < 0.01, ^∗^*p*_*FWE*_ < 0.05, ^†^*p*_*uncorr*_ < 0.05.*

The correlation analysis between the DSS score and relative activation in the processing of self-related conflicting information extracted from the GLM analysis confirmed the ROI results, revealing a cluster in dACC, *k* = 502, *p*_FWE_ = 0.01, peaking at [–12 31.5 27], *Z* = 4.22. No other suprathreshold region or trending region was identified.

#### PPI Results

The PPI analysis (**Table 3**) revealed a trend in which the dACC showed decreased functional connectivity with the left caudate during the processing of conflicting information relevant to self. Furthermore, participants’ DSS scores were positively correlated with functional connectivity during the processing of self-related conflicting information between the dACC and the left caudate, the left middle temporal gyrus, and the brainstem and hippocampus areas, all of which appeared as trends in the results.

**Table 3:** Results of Psychophysiological Interactions (PPI) analysis.

Regions	BA	*k*	Peak			
			
			*x*	*y*	*z*	*Z*
*Regions with decreased functional connectivity with dACC during the processing of conflicting information relevant to self*						
Left caudate		249^†^	–16.5	–16.5	24	4.59
*Regions whose functional connectivity with dACC during the processing of conflicting information relevant to self are associated with the DSS score*						
(+) Left caudate		74^†^	–12.5	15	13.5	4.04
(+) Left MTG	21	375^†^	–46.5	–19.5	–10.5	3.60
(+) Left brainstem/hippocampus		237^†^	–7.5	–4.5	–19.5	3.60


*MTG, middle temporal gyrus; ^†^*p*_*uncorr*_ < 0.05.*

## Discussion

In the current study, we examined the neural correlates of dialectical self-thinking by manipulating whether presented pairs of traits were contradictory or not in a modified self-referential thinking paradigm and correlated participants’ brain activity in the task with their dispositional level of naïve dialecticism. Based on the literature on self-processing and conflict monitoring, we hypothesized that, for chronic dialectical thinkers, the dACC should play a critical role in dealing with conflicting information in relation to the self. The results showed that the strength of dACC activation during the processing of conflicting information relevant to the self was strongly positively correlated with participants’ scores on the DSS. The results are in line with previous behavioral studies showing that dialectical thinking styles in various domains are mostly evident in people with high DSS scores (e.g., English and Chen, 2007; Spencer-Rodgers et al., 2009, 2004). We extended this behavioral work by showing that for the chronic dialectical thinkers, the dACC was more engaged when processing conflict information in relation to the self. It is well known that the engagement of the ACC has been observed in studies of self-thinking (e.g., Northoff and Bermpohl, 2004; Northoff et al., 2006; Zhu et al., 2007), and the effect is maintained even after controlling for confounding effects of familiarity (Qin et al., 2012). On the other hand, the dACC has been reported to play a crucial role in conflict-monitoring (Botvinick et al., 2004; Carter and van Veen, 2007). Since dialectical self-thinking is a specific form of self-processing that deals with conflicting information relevant to self, the enhanced activity in the dACC in dialectical self-thinking may reflect more efficient monitoring and processing of self-related information compared with information relating to other under conditions of contradiction.

All of our participants were Chinese, who are supposed to be more likely to be dialectical thinkers (Peng and Nisbett, 1999), hence one might expect that there should also be an overall interaction effect between judgment target and trait type on dACC’s activity regardless of the DSS score. However, previous research has shown that there are considerable and meaningful individual differences in naïve dialecticism even within a single culture (Spencer-Rodgers et al., 2004, 2009; English and Chen, 2007; Boucher et al., 2009), and in our study the DSS score of the low DSS group (*M* = 3.91) was, in fact, closer to the results for Western participants in previous research (*M* = 3.87, Hamamura et al., 2008). Since dialectical self-thinking, by its definition, should only occur with individuals possessing a dialectical thinking style, it is not surprising that our diverse sample did not show an overall significant interaction effect in the dACC.

A closer look into the DSS’s positive correlation with the dACC’s relative activation in self-related conflict-processing, however, revealed an unexpected effect: for the low DSS group: The dACC showed enhanced activity during self- vs. other-referential processing on non-contradictory trials. The non-contradictory condition served as a baseline in the current study and was essentially a classical self-referential task. Therefore, it is possible that this effect reflects a more general self-referential process. As participants’ dispositional level of naïve dialecticism increased, the dACC’s function might shift from general self-processing to a more specialized role in dealing with conflicting aspects of the self. However, this explanation is highly speculative and needs to be tested in future research.

Another unexpected result was the lack of DSS correlation at behavioral level. While previous studies showed that a higher score on DSS is associated with more dialectical ratings to contradictory adjective pairs (Spencer-Rodgers et al., 2004, 2009), such positive correlations were not observed in the current study. This inconsistency might be attributed to the simplified ‘yes’–‘no’ response employed in the current study. While dialectical thinkers are more likely to feel that contradictory adjectives may simultaneously describe themselves, due to dialecticism’s emphasis on finding the middle ground, they are also less liable to choose an extreme response on a rating scale (e.g., choose 5 or 6 instead of 7 on a 7-point Likert-like scale; Hamamura et al., 2008). A ‘yes’–‘no’ response might be just not sensitive enough to capture dialectical responses. Future studies could try to employ Likert-like rating scales to measure dialectical self-thinking at behavioral level.

The view of dACC’s critical role in dialectical self-thinking is also consistent with the results in the PPI analysis. For the chronic dialectical thinkers, when dealing with conflicting information relevant to self, there was increased functional connectivity between the dACC and a number of self-related regions, including the caudate, which has been linked with processing of self-relevant information as well as reward (e.g., Enzi et al., 2009), and the middle temporal gyrus, which has been linked with context-dependent retrieval of episodic memory (e.g., Tsukiura et al., 2002) and self-referential processing (e.g., Yoshimura et al., 2009). These results may reflect the use of the dACC by dialectical thinkers in modulating and resolving contradiction in self-processing, enhancing its functional connectivity with other self-processing regions. However, it should be noted that these regions showed only significant trends, and more studies are needed to verify them. Overall, this study provides the first evidence that the dACC is engaged in dialectical thinking in the domain of self. Additional theoretical and empirical studies could try to elaborate the dACC’s role in dialectical self-processing (e.g., monitoring, resolution, or integration of conflicting self-related information) and delineate the underlying neural network.

Beyond the results in the dACC, exploratory whole-brain analysis further identified the left hippocampus and parahippocampus as being engaged during the processing of conflicting information relevant to self regardless of participants’ dispositional tendency to think dialectically. These regions are well known as key areas in an autobiographical memory (AM) network (Svoboda et al., 2006; Addis et al., 2012). The engagement of these regions may reflect participants’ effort in retrieving autobiographical memories in relation to personality traits during self-referential judgments, but further work is required to verify this.

Although our sample was relatively distributed on the level of dispositional dialectical thinking, they were still from a single culture and shared the same cultural and societal environment. Since most existing findings regarding dialectical thinking come from cross-cultural studies, it would also be interesting to replicate the current study in a cross-cultural comparison between East-Asian and Western participants to see if activity in the dACC does indeed underlie the cultural differences in dialectical thinking. In addition, the tendency of participants to think dialectically can be temporarily altered through priming (e.g., Ma-Kellams et al., 2011, Study 3), to make a stronger causal inference based on this intervention. It is expected that the enhanced activity in the dACC during the processing of self-related conflicting information should be particularly pronounced for those primed with a dialectical mindset.

## Author Contributions

FW developed the study concept with KP and JS. FW developed the experimental paradigm with KP, JS and YB. FW collected the data. FW performed the data analysis and interpretation under the supervision of JS. FW and JS drafted the manuscript. All authors contributed to discussion of the manuscript. JS, PS, YZ and PR provided critical revisions. All authors approved the work for publication.

## Conflict of Interest Statement

The authors declare that the research was conducted in the absence of any commercial or financial relationships that could be construed as a potential conflict of interest.

## Footnotes

1 In previous studies, both contradiction in trait meaning (e.g., Spencer-Rodgers et al., 2009, Study 2) and contradiction in valence (e.g., Spencer-Rodgers et al., 2004, Study 1) have been used to operationally define of the dialectical self. This was partly reflects the fact that contradiction in meaning and valence are neutrally confounded (Spencer-Rodgers et al., 2009). In the current study, these two factors were bound together to represent the higher-level construct of the dialectical self.
